# Effect of dietary defatted diatom biomass on egg production and quality of laying hens

**DOI:** 10.1186/2049-1891-5-3

**Published:** 2014-01-09

**Authors:** Xiangjun Leng, Kun-Nan Hsu, Richard E Austic, Xin’ gen Lei

**Affiliations:** 1 Department of Animal Science, Cornell University, Ithaca, NY 14853, USA; 2 College of Aquaculture and Life Science, Shanghai Ocean University, Shanghai 201306, China

**Keywords:** Defatted diatom algae, Egg, Health, Laying hen, Soybean

## Abstract

**Background:**

This study was to determine if feeding laying hens with defatted diatom microalgal biomass (DFA) from biofuel production affected their egg production and health status.

**Methods:**

Five replicates of 5 individually caged ISA Babcock White leghorn hens were fed 4 diets, including a corn-soybean meal control diet, a diet containing 7.5% DFA substituting for soybean meal, and diets containing 7.5% or 15% DFA substituting for corn and soybean meal. Body weights, feed intake, feed conversion ratio (FCR), rate of egg production, egg size, egg mass, and several characteristics of eggs were determined at 4 and 8 wk. Venous blood was sampled at 4 and 8 wk for measurement of 5 biomarkers of health.

**Results:**

The 15% DFA diet decreased (*P <* 0.05) feed intake, egg production, and plasma uric acid concentrations as compared with the control diet, but increased (*P <* 0.05) egg albumen weight and height compared with the 7.5% DFA diets. The two levels of DFA produced dose-dependent (*P <* 0.05) changes in three color measures of egg yolk, without affecting four hen plasma biochemical indicators of health.

**Conclusions:**

Feeding laying hens with 7.5% DFA in the corn-soybean meal diet for 8 wk had no adverse effect on their health, egg production, or egg quality, but 15% inclusion reduced feed intake, egg production, and efficiency of feed utilization.

## Background

Although fossil fuels are the major source of energy for heating, transportation, manufacturing, and the generation of electricity, these fuels are non-renewable. Therefore, the search for renewable energy sources has become a key challenge of this century. Microalgae are recognized as one of the oldest living micro-organisms, and are seen as a very attractive source of renewable biofuels, especially biodiesel. Many species of microalgae contain large amounts of lipids that are suitable for the production of biofuels [1]. Microalgae are the natural food source for many important aquaculture species such as molluscs, shrimps and fish [2]. Several species of microalgae have been reported to be acceptable for inclusion in diets for swine, rabbits, broiler chickens, laying hens, and ruminant animals [3,4]. Soybean meal is a by-product of the extraction of oil from soybeans. With high protein content, it is rich in essential amino acids and serves as the main protein source for food-producing animals. Due to the increased human demand for soybean products, soybean meal is becoming more expensive and limited in supply [5,6]. Therefore, it is important to explore other high protein feedstuffs for the sustainable development of animal production.

Microalgae are a rich source of protein, essential fatty acids, vitamins and minerals [3]. After lipid removal, the residual biomass contains even higher concentrations of protein and other nutrients. Microalgae are good sources of long chain polyunsaturated fatty acids (PUFA) and have been used to enrich diets with omega-3 PUFA [7-9]. Diatoms are a class of microalgae that characteristically accumulate amorphous silicon in their membranes, resulting in distinct external features called frustules [10]. The defatted diatom biomass contains protein, residual fat, carbohydrates, silicon and a large mineral fraction that includes calcium, phosphorus, sodium, potassium, chloride, and several other nutritionally significant minerals [3,11-13]. At the present time, a diatom species, *Staurosira sp*, is under investigation as a lipid source for biofuel production. Apparently, economics of that attempt would be remarkably enhanced if its defatted biomass is used as a feedstuff. However, no such study has been reported on feeding values of the defatted biomass of *Staurosira sp* or any diatom species in diets for poultry or swine. The frustules structure and high ash content in the defatted biomass could be a practical constraint for feed application. With a limited amount of defatted *Staurosira sp* microalgal biomass available for testing, we conducted the present laying hen experiment for 8 wk. Our objectives were to determine if the defatted biomass could partly replace soybean meal or a combination of corn and soybean meal in a practical diet; and how the replacements affected feed intake, health status, and egg production of the hens, and biochemical and biophysical properties of their eggs including pigmentation of yolk.

## Materials and methods

### Animals, dietary treatments, and management

ISA Babcock White Leghorn laying hens (n = 100, 47 wk old, *Gallus gallus domesticus*), with an initial body weight of 1.57 ± 0.20 kg, were randomly assigned to 4 dietary treatments. There were 5 replicates for each treatment and each replicate consisted of 5 individually-caged hens. The cages with a size of 0.44 m high × 0.30 m wide × 0.45 m deep were equipped with nipple drinkers and trough feeders. The hen-house was provided with 16 h light per day and the hens were given free access to feed and water. The duration of the experiment was 8 wk. The animal protocol was approved by the Institutional Animal Care and Use Committee at Cornell University.

Defatted *Staurosira sp* microalgal biomass (DFA) in the form of powder was generated from the research on algal cultures for biofuel production (Cellana, Kailua-Kona, HI). Proximate and mineral analyses of the defatted alga were done by Dairy One, Inc. (Ithaca, NY) and amino acid analyses were performed by Experiment Station Chemical Laboratories (University of Missouri, Columbia, MO). The composition of the defatted alga is presented in Table 1. The four experimental diets included a corn-soybean meal control diet, the diet with 7.5% DFA and the 5 most limiting amino acids (Lys, Met, Ile, Thr, Trp) substituting for 7.5% soybean meal (7.5% algae-SBM), and two diets with DFA substituting for 7.5% (7.5% algae-SBM/Corn) or 15% (15% algae-SBM/Corn) soybean meal and corn (about 1:3.1). The same source of algae was used for all diets. The DFA had a similar crude protein level and amino acids composition as the mixture of soybean meal and corn with a ratio of 1:3.1. All diets were formulated to be isocaloric (2.80 Mcal of ME/kg), and iso-Ca, and iso-P by adjusting vegetable oil, dicalcium phosphate and limestone. The DFA contained many other minerals, but the dietary levels were not adjusted because the objective of the experiment was to test the acceptability of the algae, with its high ash content, as a feed ingredient. The compositions and nutrient concentrations of the diets are presented in Table 2.

**Table 1:** **Chemical composition of the defatted diatom microalgal biomass**^**1**^

**Item**	**Content**	**Item**	**Content**
ME, Kcal/g	1.32	Met	0.33
Protein	19.1	Cys	0.32
Fat	3.3	Gly	0.96
Fiber	14.7	Ser	0.76
Ash	44.9	His	0.30
Moisture	6.9	Ile	0.78
Ca	2.78	Leu	1.33
P	0.76	Phe	0.86
Na	3.94	Tyr	0.57
K	1.66	Thr	0.88
Cl	6.34	Trp	0.18
Arg	0.93	Val	0.98
Lys	0.83		

^1^All items except ME are % of biomass (as fed basis).

**Table 2:** Compositions and nutrient concentrations of experimental diets

**Ingredient,% as fed**	**Control**	**7.5% Algae**	**7.5% Algae**	**15% Algae**
		**-SBM**	**-SBM/Corn**	**-SBM/Corn**
Corn (yellow)	65.65	65.28	59.45	52.55
Vegetable oils	0.55	1.55	2.30	4.30
Soybean meal	22.50	15.00	20.50	18.50
(48.5% crude protein)				
Dicalcium phosphate	1.50	1.40	1.35	1.20
Limestone	8.80	8.40	8.40	7.95
Salt	0.50	0.00	0.00	0.00
Vitamin mix^1^	0.25	0.25	0.25	0.25
Mineral mix^2^	0.15	0.15	0.15	0.15
DL-methionine	0.10	0.10	0.10	0.10
Defatted algae	0.00	7.50	7.50	15.00
Amino acids premix^3^	0.00	0.37	0.00	0.00
Total	100.00	100.00	100.00	100.00
Nutritional composition (% except for ME)				
ME, Kcal/g	2.80	2.80	2.80	2.80
Protein	16.49	14.26	16.43	16.30
Fat	3.27	4.73	5.31	7.58
Fiber	2.32	3.12	3.21	4.08
Ca	3.75	3.76	3.76	3.76
P	0.60	0.59	0.60	0.60
Avail. P	0.38	0.40	0.40	0.42
Arg	1.03	0.86	1.03	1.02
Lys	0.84	0.84	0.85	0.85
Met	0.36	0.36	0.38	0.39
Cys	0.28	0.23	0.26	0.23
Gly	0.68	0.61	0.70	0.73
Ser	0.80	0.68	0.80	0.79
His	0.44	0.36	0.42	0.40
Ile	0.67	0.67	0.68	0.69
Leu	1.50	1.33	1.48	1.46
Phe	0.78	0.67	0.78	0.78
Tyr	0.64	0.55	0.64	0.64
Thr	0.61	0.61	0.63	0.65
Trp	0.21	0.21	0.21	0.22
Val	0.76	0.68	0.78	0.79

^1^Provided (in IU/kg of diet): vitamin A, 6,500; vitamin D_3_, 3,500; vitamin E, 25 and (in mg/kg of diet): riboflavin, 25; d-calcium pantothenate, 25; nicotinic acid, 150; cyanocobalamin, 0.011; choline chloride, 1,250; biotin, 1.0; folic acid, 2.5; thiamine hydrochloride, 7.0; pyridoxine hydrochloride, 25.0; menadione sodium bisulfite, 5.0; and ethoxyquin, 66.

^2^Provided (in mg//kg of diet): CuSO_4_^.^5H_2_O, 31.42; KI, 0.046; FeSO_4_^.^7H_2_O, 224.0; MnSO_4_^.^H_2_O, 61.54; Na_2_SeO_3_, 0.13; ZnO, 43.56; Na_2_MoO_4_^.^2H_2_O, 1.26.

^3^Provided 1.4 g Lys, 0.1 g Met, 0.9 g Ile, 0.6 g Thr, 0.3 g Trp per kilogram of diet.

### Sample collection and procedures

Body weights of the laying hens were recorded at the start and the end of the experiment. Eggs were collected daily and egg production was calculated on a hen-day basis. Feed intake and egg production was recorded weekly by replicates to calculate feed conversion ratio (FCR). Eggs produced on the last 3 d of the 4^th^ wk and 8^th^ wk were individually weighed and analyzed for their interior and exterior quality. Eggs were examined for shell quality by measuring egg shell thickness, breaking strength, and egg specific gravity. Shell thickness, without shell membrane, was measured by micrometer with a mean value of measurements at 3 locations on the eggs (near the equator). The strength of egg shells was determined as the compression pressure necessary to crack the shell when the egg was placed horizontally between the plates of an Instron model 5969 (Instron, Norwood, MA). The specific gravity of eggs was determined by the buoyancy of eggs in salt solutions of varying density. Egg components, including albumen, yolk and shell, were weighed separately. Haugh units, a measure of the height of the albumen of eggs broken out on a flat surface were determined by the use of a micrometer [14]. Yolk color, measured as L^*^-, a^*^- and b^*^-values, was determined with a Macbeth Color Eye (Macbeth Division of Kollmorgen Instruments Corp. Newburgh, NY). The L* value represents lightness (negative towards black, positive towards white), the a* value red–greenness (negative towards green, positive towards red) and the b* value the blue–yellow color scale (negative towards blue, positive towards yellow).

Uric acid, alanine transaminase (ALT), cholesterol and glucose in plasma were determined with Uric acid liquid stable reagent (Infinity TM, Fisher Diagnostics, Fisher Scientific Company, LLC, Middletown, NY), ALT Liquid stable reagent (Thermo Electron Corporation, Pittsburgh, PA), Cholesterol reagent (Wako Pure Chemical Industries, Ltd, Osaka, Japan), and Glucose Assay Kit (Sigma, St Louis, MO), respectively. Alkaline phosphatase (AKP) was determined by the method of Bowers and McComb [15]. Yolk cholesterol was extracted by the method of Folch *et al.*[16] and measured by the HPLC method of Beyer and Jensen [17].

### Statistical analysis

The experimental data were presented as mean ± standard error, using SPSS17.0 for one-way analysis of variance, combined with Duncan’s method for multiple comparisons. The significance level for differences was *P <* 0.05.

## Results

### Body weight, feed intake, egg production rate, and biochemical indices of hens

There were no significant differences in body weight among treatment groups at the beginning or the end of the experiment, despite an average loss of 20 to 70 g/hen during the experiment across all groups (Table 3). In the first 4 wk, the inclusion of defatted algae tended to decrease feed intake, egg production rate, egg mass, and FCR. In the second 4 wk period, hens fed 15% DFA had lower (*P* < 0.05) feed intake, egg production rate, egg mass, and higher (*P <* 0.05) FCR than those of the control group. During the whole 8-wk period, hens fed 15%, but not 7.5% DFA had lower (*P* < 0.05) egg production rate (−12%), daily feed intake (− 9 g/hen/d) and higher (*P* < 0.05) FCR (+0.09 g feed/g egg) than those fed the control diet (Table 3). There were no significant differences in plasma AKP, ALT, cholesterol, or glucose among dietary groups at the end of study (the 8^th^ wk) (*P >* 0.05) (Table 4). However, hens fed 15% DFA had lower (*P* < 0.05) plasma uric acid level than that of control group.

**Table 3:** Effect of defatted diatom microalgal biomass on body weight, feed intake, egg production rate, egg mass and FCR

**Item**	**Control**	**7.5% Algae**	**7.5% Algae**	**15% Algae**
		**-SBM**	**-SBM/Corn**	**-SBM/Corn**
Body weight, kg				
Wk 0	1.53 ± 0.16	1.60 ± 0.19	1.58 ± 0.18	1.58 ± 0.20
Wk 8	1.47 ± 0.08	1.58 ± 0.08	1.53 ± 0.11	1.51 ± 0.10
Daily feed intake, g/hen				
Wk 1-4	100.1 ± 4.9	96.9 ± 3.9	95.9 ± 6.7	92.6 ± 11.2
Wk 5-8	96.0 ± 8.3^a^	96.0 ± 3.6^a^	95.0 ± 6.9^a^	85.5 ± 4.5^b^
Wk 1-8	98.1 ± 6.0^a^	96.4 ± 3.4^ab^	95.4 ± 5.9^ab^	89.1 ± 5.9^b^
Egg production rate, %^1^				
Wk 1-4	86.7 ± 3.4	83.7 ± 6.3	84.7 ± 6.6	75.1 ± 16.4
Wk 5-8	81.3 ± 11.2^a^	85.6 ± 5.0^a^	83.9 ± 8.7^a^	69.0 ± 6.6^b^
Wk 1-8	84.0 ± 7.1^a^	84.6 ± 4.7^a^	84.3 ± 7.1^a^	72.1 ± 10.1^b^
Egg mass, g/hen/d				
Wk 1-4	52.02 ± 2.02	49.79 ± 3.68	50.21 ± 3.23	45.63 ± 8.65
Wk 5-8	48.77 ± 5.02^a^	49.73 ± 2.23^a^	49.71 ± 4.34^a^	41.43 ± 4.01^b^
Wk 1-8	50.40 ± 3.87^a^	49.74 ± 2.93^a^	49.96 ± 3.36^a^	43.53 ± 5.71^b^
FCR^2^				
Wk 1-4	1.92 ± 0.03	1.95 ± 0.05	1.91 ± 0.06	2.02 ± 0.11
Wk 5-8	1.97 ± 0.08^a^	1.93 ± 0.04^a^	1.92 ± 0.05^a^	2.06 ± 0.05^b^
Wk 1-8	1.94 ± 0.06^a^	1.94 ± 0.04^a^	1.92 ± 0.05^a^	2.05 ± 0.09^b^

^a,b^Means in the same row without a letter in common are different (*P <* 0.05).

^1^Egg production = 100 [number of eggs laid ÷ (number of hens x number of days)].

^2^FCR = feed intake (g) / egg mass (g).

**Table 4:** **Effect of defatted diatom microalgal biomass on plasma biochemical indices of hens at the 8**^**th **^**wk**

**Item**	**Control**	**7.5% Algae**	**7.5% Algae**	**15% Algae**
		**-SBM**	**-SBM/Corn**	**-SBM/Corn**
AKP, U/mL	111.5 ± 29.0	146.4 ± 34.1	155.4 ± 44.2	133.8 ± 31.1
ALT, U/L	24.1 ± 6.5	24.3 ± 9.1	33.5 ± 15.8	29.5 ± 7.4
Cholesterol, mg/dL	117.5 ± 8.5	117.4 ± 12.3	112.6 ± 22.5	106.0 ± 15.6
Glucose, mg/dL	204.9 ± 4.6	210.7 ± 13.4	209.1 ± 9.4	198.6 ± 7.0
Uric acid, mg/dL	10.7 ± 1.9^a^	10.2 ± 2.7^ab^	8.6 ± 2.4^ab^	7.6 ± 1.3^b^

^a,b^Means in the same row without a letter in common are different (*P* < 0.05).

### Egg quality and yolk color

At the end of 4^th^ and 8^th^ wk, there were no significant differences between the control and the DFA diets in egg quality indexes (Table 5), including egg weight, Haugh unit, yolk weight, shell weight, albumen weight, and shell thickness. However, the 15% Algae-SBM/Corn diet had higher (*P* < 0.05) egg albumen weight and height than the 7.5% Algae-SBM and 7.5% Algae-SBM/Corn diets at the 8^th^ wk. Additionally, the DFA diets produced no difference from the control diet in egg specific gravity, egg shell breaking strength or yolk cholesterol at the end of study (the 8^th^ wk). Including DFA in the diets affected (*P* < 0.05) yolk color in a dose-dependent fashion (Table 6). The L^*^ (lightness) value and + b^*^ (yellowness) value of yolk decreased (*P* < 0.05) with the increasing DFA of the diet. At the 8^th^ wk, but not at the 4^th^ wk, the values for lightness and yellowness were lower (*P* < 0.05) for 7.5% algae-SBM/Corn than for 7.5% algae-SBM. The + a^*^ (redness) values of yolk were elevated (*P* < 0.05) by all three DFA diets at the end of 4^th^ wk, but by only the two 7.5% DFA diets at the 8^th^ wk.

**Table 5:** **Effect of defatted diatom microalgal biomass on egg quality at the 4**^**th **^**and 8**^**th **^**wk**

**Item**	**Control**	**7.5% Algae**	**7.5% Algae**	**15% Algae**
		**-SBM**	**-SBM/Corn**	**-SBM/Corn**
At the end of the 4^th^ wk				
Egg weight, g	60.00 ± 3.93	59.49 ± 5.25	59.28 ± 3.79	60.69 ± 3.61
Haugh unit	86.6 ± 4.0	84.6 ± 6.4	85.6 ± 7.1	88.6 ± 5.7
Yolk weight, g	15.79 ± 1.42	16.35 ± 1.42	15.77 ± 1.22	15.92 ± 0.82
Egg shell weight, g	5.29 ± 0.57	5.70 ± 2.11	5.37 ± 0.39	5.60 ± 0.54
Albumen weight, g	38.81 ± 2.96	38.21 ± 3.89	38.13 ± 2.83	39.00 ± 3.24
Egg shell thickness, mm	0.34 ± 0.03	0.34 ± 0.03	0.36 ± 0.03	0.36 ± 0.03
At the end of the 8^th^ wk				
Egg weight, g	59.99 ± 2.51	58.09 ± 1.49	59.25 ± 1.75	60.05 ± 1.26
Haugh unit	82.6 ± 3.1	82.0 ± 3.3	81.6 ± 2.0	84.6 ± 1.4
Yolk weight, g	16.11 ± 0.71	16.25 ± 0.55	16.08 ± 0.48	15.90 ± 0.32
Egg shell weight, g	5.47 ± 0.26	5.28 ± 0.25	5.38 ± 0.25	5.49 ± 0.35
Albumen weight, g	38.44 ± 2.17^ab^	36.78 ± 1.40^a^	37.87 ± 1.45^ab^	39.24 ± 1.15^b^
Albumen height, mm	6.92 ± 0.43^ab^	6.73 ± 0.47^ab^	6.71 ± 0.24^a^	7.23 ± 0.23^b^
Egg shell thickness, mm	0.35 ± 0.02	0.35 ± 0.01	0.34 ± 0.03	0.35 ± 0.02
Egg specific gravity	1.082 ± 0.004	1.080 ± 0.001	1.080 ± 0.005	1.080 ± 0.002
Egg shell strength, N	34.4 ± 2.2	34.4 ± 2.0	32.6 ± 4.6	35. 3 ± 3.6
Yolk cholesterol, mg/g	14.20 ± 0.66	14.06 ± 0.34	13.50 ± 0.66	13.69 ± 0.71

^a,b^Means in the same row without a letter in common are different (*P <* 0.05).

**Table 6:** Effect of defatted algae on yolk color

**Item**	**Control**	**7.5% Algae**	**7.5% Algae**	**15% Algae**
		**-SBM**	**-SBM/Corn**	**-SBM/Corn**
At the end of the 4^th^ wk				
L^*^(lightness)	54.806 ± 0.541^a^	52.502 ± 0.602^b^	51.943 ± 0.650^b^	50.328 ± 0.878^c^
+a^*^(redness)	11.576 ± 0.209^a^	13.438 ± 0.305^b^	13.316 ± 1.015^b^	12.939 ± 0.329^b^
+b^*^(yellowness)	35.660 ± 0.314^a^	34.522 ± 0.320^b^	34.013 ± 0.383^b^	33.038 ± 0.558^c^
At the end of the 8^th^ wk				
L^*^(lightness)	54.758 ± 0.430^a^	52.718 ± 0.393^b^	51.690 ± 1.014^c^	50.732 ± 0.762^d^
+a^*^(redness)	11.084 ± 0.317^a^	12.573 ± 0.288^b^	12.518 ± 0.889^b^	11.356 ± 0.607^a^
+b^*^(yellowness)	35.479 ± 0.238^a^	34.499 ± 0.149^b^	33.697 ± 0.561^c^	32.856 ± 0.239^d^

^a,b,c,d^Means in the same row without a letter in common are different (*P* < 0.05).

## Discussion

Results of the present study indicate that 7.5% DFA of *Staurosira sp* could replace 7.5% soybean meal or a combination of corn and soybean meal in diets for laying hens, without adverse effects on their body weights, feed intake, FCR, health status, or egg mass, egg production rate and quality. However, the inclusion of 15% DFA decreased hen feed intake by 11% and egg production rate by 12%. Past studies on inclusions of algae in diets for poultry have shown that algae can be used safely at dietary levels of 5% to 10% [18-24]. Thus, our finding on 7.5% DFA as a tolerable level for hens just falls into the middle of these studies. The decreases in feed intake and egg production rate in the 15% DFA group were likely attributed to the high ash (44.9%) and sodium chloride (10.3%) concentrations of the DFA used in the study. Although we removed salt and decreased limestone and dicalcium phosphate supplementation in the DFA diets, the 15% inclusion rate rendered the diet still salty and probably less palatable than the control diet. As expected, hens fed this diet were observed to drink more water and have bulky wet droppings. While the hens seemed to tolerate 7.5% DFA in the diet well for 8 wk, effects of long-term ingestion of high ash and salt on the health and egg production of these animals and on moisture and bulk of their excreta and the subsequent manure disposal should be evaluated in future research.

Inclusion of 7.5 and 15% DFA resulted in dose-dependent changes of egg yolk color, but did not significantly affect egg weights, egg shell weight, egg shell thickness, egg breaking strength, albumen firmness (Haugh units), or yolk cholesterol concentrations. While the latter outcome is consistent with other reports involving chickens [21,22,24-27] and Japanese quail [22], in which one or more of these traits were reported, the former is likely due to the presence of various carotenoids in DFA. Although algae powder appears as a bluish-green color, it contains high levels of carotenoids such as β-carotene, lutein and zeaxanthin [28]. The red carotenoid astaxanthin is present in some algae [2]. Because poultry and other animals cannot synthesize carotenoids *de novo*, the color of broiler skin and leg and egg yolk can be enhanced by including algae in feeds [3]. In a study using a color meter to measure yolk pigmentation [29], 2.4% or 4.8% of a commercial preparation of the marine algae, *Schizochytrium*, was fed to Leghorn hens over 4 wk. The yolk redness (+a* value) was increased, and the yellowness (+b* value) and lightness (+L* value) were decreased in a dose-responsive manner to the algae. In another study, 10% and 20% algae (*Nannochloropsis oculata*) in diet increased the yolk +a^*^ value, and decreased the +L* value [30]. Consistently, the present study showed DFA dose-dependent increases in the + a value and decreases in the +b and +L* values of yolk. These color changes are supposed to be highly correlated with the Roche color fan that is used to measure the pigmentation in egg yolks and poultry skin by visual examination. Increases in the numerical color fan score have been observed in various studies on the use of algae in diets of laying hens [9,24,27,30,31]. Depending on the amount of algae or lipid extract of algae incorporated into wheat- and sorghum-based feeds, scores ranged from about 7 to 9, which are considered acceptable for table eggs, to 12 to 15 which are in the range suitable for manufactured food products such as egg noodles and bakery items. Increases in yolk pigmentation were reported to occur within 24 h and to reach plateau at 7 to 8 d [9,31]. In the present study, we found that 7.5 or 15% DFA shifted yolk pigmentation score from 7.9 in the control group to 9.3 to 9.5 (data not shown). This shift implies that DFA may be used as a source of pigments for egg yolk formation to improve its consumers’ acceptance.

Several genera of algae have been analyzed for protein quality based on protein digestibility, biological value, and net protein utilization [3,4]. Although the results indicate that protein quality varies among algae, many species have relatively high protein content and excellent protein quality by these measures. In the present experiment, DFA was added to the diet in replacement of an equivalent weight of soybean meal or a mixture of soybean and corn. The DFA contained 19% crude protein in contrast to 48% crude protein in soybean meal. Substitution of one-third of the soybean meal with DFA necessarily means that the resulting diets had less protein than the control diet. Several amino acids were added to the 7.5% Algae-SBM diet in an attempt to meet essential amino acid requirements. This diet supported production responses that were similar to those of the control diet. Only uric acid, among the plasma biomarkers, was affected by the DFA treatments. The lower plasma uric acid concentration in the hens fed 15% defatted diatom suggests that less amino acid catabolism had occurred in this group of hens as compared to the control groups. A simple explanation for this observation would be that the 11% lower daily feed (and protein) consumption resulted in fewer amino acids available for catabolism. Alternatively or additionally, a low digestibility of the protein in the 15% DFA diet may have contributed to reduced absorption of amino acids, thereby resulting in lower amino acid catabolism. Additional research would be necessary to determine the actual cause of the reduction in plasma uric acid concentration.

Due to the limited amount of DFA available for the present study, our feeding experiment lasted for only 8 wk. However, all responses of feed intake, egg production, egg quality, and plasma biomarkers were consistent that DFA could serve at a dietary level of 7.5% over this period as a source of protein, energy, calcium, phosphorus, and sodium chloride for laying hens. The question is if the period of 8 wk was sufficient for the objectives of this experiment including determining effects of the DFA nutrients on egg production and composition. Physiologically, the formation of egg yolk occurs in a process that lasts about 9 d. Egg albumen, eggshell membranes, and eggshell are usually secreted within slightly more than 24 h. Experimentally, changes in yolk pigmentation occurred within 8 d after the addition of algae to the diet [9,31]. Changes in egg production occurred within 2 to 3 mo of the feeding protein- or amino acid-deficient diets to laying hens [32,33]. Concentrations of selenium or fat-soluble vitamins in eggs were affected by diet within days to a few weeks [34-36]. Clearly, many nutritional effects on egg production and composition could be detected within 8 wk. In fact, we observed significant effects of DFA on egg yolk color measures after only 4-wk feeding. Metabolically, animals should be most responsive during the initial phase to negative impacts of DFA as longer time feeding may give them chance to adapt. Nevertheless, it is prudent that a feeding trial of several months or a full year would be necessary to evaluate long-term nutritional values of DFA during a full egg production cycle.

## Competing interests

The authors declare that they have no competing interests.

## Authors’ contributions

XGL and REA conceived the study, supervised the trial, and revised the manuscript. XL, K-NH, and REA raised the animals and carried out the laboratory analysis. XL and REA drafted the initial manuscript, and XGL helped revise and finalize the manuscript. All authors read and approved the final manuscript.
